# Comparison of Maternal and Fetal Outcomes in Parturients With and Without a Diagnosis of Gestational Diabetes

**DOI:** 10.1055/s-0039-1696947

**Published:** 2019-11

**Authors:** Inês Carolina Siqueira Freitas, Micheli Cristiane Hintz, Larissa Chaiane Orth, Tamara Gonçalves da Rosa, Betine Moehlecke Iser, Carine Psendziuk

**Affiliations:** 1 Medical School, Universidade do Sul de Santa Catarina, Tubarão, SC, Brazil; 2 Postgraduate Program in Health Sciences, Universidade do Sul de Santa Catarina, Tubarão, SC, Brazil; 3 Universidade do Sul de Santa Catarina, Tubarão, SC, Brazil

**Keywords:** gestational diabetes, hyperglycemia, high-risk pregnancy, macrosomia, diabetes gestacional, hiperglicemia, gravidez de alto risco, macrossomia

## Abstract

**Objective** The present study aims to compare the maternal and fetal outcomes of parturients with and without a gestational diabetes diagnosis.

**Methods** A case-control study including parturients with (cases) and without (control) a gestational diabetes diagnosis, who delivered at a teaching hospital in Southern Brazil, between May and August 2018. Primary and secondary data were used. Bivariate analysis and a backward conditional multivariate logistic regression were used to make comparisons between cases and controls, which were expressed by odds ratio (OR), with a 95% confidence interval (95%CI) and a statistical significance level of 5%.

**Results** The cases (*n* = 47) were more likely to be 35 years old or older compared with the controls (*n* = 93) (*p* < 0.001). The cases had 2.56 times greater chance of being overweight (*p* = 0.014), and a 2.57 times greater chance of having a positive family history of diabetes mellitus (*p* = 0.01). There was no significant difference regarding weight gain, presence of a previous history of gestational diabetes, height, or delivery route. The mean weight at birth was significantly higher in the infants of mothers diagnosed with diabetes (*p* = 0.01). There was a 4.7 times greater chance of macrosomia (*p* < 0.001) and a 5.4 times greater chance of neonatal hypoglycemia (*p* = 0.01) in the infants of mothers with gestational diabetes.

**Conclusion** Therefore, maternal age, family history of type 2 diabetes, obesity and pregestational overweightness are important associated factors for a higher chance of developing gestational diabetes.

## Resumo

**Objetivo** O presente estudo tem como objetivo comparar os desfechos maternos e fetais das parturientes com e sem diagnóstico de diabetes gestacional.

**Métodos** Estudo caso-controle, incluindo parturientes com (casos) e sem (controle) diagnóstico de diabetes gestacional, que tiveram parto em um hospital de ensino no Sul do Brasil, entre maio e agosto de 2018. Foram utilizados dados primários e secundários. Análise bivariada e regressão logística multivariada condicional retrógrada foram utilizadas para fazer comparações entre casos e controles, expressas por razão de probabilidades (RP), com intervalo de confiança de 95% (IC95%) e nível de significância estatística de 5%.

**Resultados** Os casos (*n* = 47) tiveram maior chance de ter idade superior a 35 anos em comparação com os controles (*n* = 93) (*p* < 0,001), chance 2,56 vezes maior de estarem acima do peso (*p* = 0,014), e chance 2,57 vezes maior de terem história familiar positiva de diabetes mellitus (*p* = 0,01). Não houve diferença significativa relacionada ao ganho de peso, história pregressa de diabetes gestacional, estatura ou via de parto. O peso médio ao nascer foi significativamente maior nos lactentes de mães com diabetes gestacional (*p* = 0,01). Houve 4,7 vezes maior chance de macrossomia (*p* < 0,001), e 5,4 vezes maior chance de hipoglicemia neonatal (*p* = 0,01) em lactentes de mães com diabetes gestacional.

**Conclusão** Portanto, idade materna, história familiar de diabetes tipo 2, obesidade e excesso de peso pré-gestacional são importantes fatores associados a uma maior chance de desenvolvimento de diabetes gestacional.

## Introduction

Diabetes mellitus (DM) is a group of metabolic diseases characterized by hyperglycemia resulting from insulin deficiency. Its incidence has been increasing over the years. The number of adults with DM worldwide increased from 30 million in 1985 to 135 million in 1995, and to 173 million in 2002, and currently this number is ∼ 415 million. In women, when detected for the first time during pregnancy, this condition is classified as gestational diabetes (GD), which is considered an important risk factor for the future development of type 2 DM (T2DM), and has a prevalence of 1–37.7%, with a worldwide average of 18%.^1^ ^2^

The risk factors for the development of GD should be evaluated in each pregnant woman so that the diagnosis can be established prematurely, enabling an adequate and early treatment. Some of the factors associated with the development of GD are overweightness, obesity or excessive weight gain during pregnancy, family history of T2DM in a first-degree relative, previous history of GD, hypertension, or preeclampsia in the current pregnancy.^3^

During pregnancy, insulin resistance increases due to the release of diabetogenic placental hormones. Pregnant women with GD have higher insulin resistance than pregnant women without GD; therefore, the postprandial glycemic values in these pregnant women are even higher. The complications of GD include fetal macrosomia, birth injury, increased rates of cesarean sections, neonatal hypoglycemia, neonatal respiratory distress syndrome (RDS), prematurity, and fetal death. Pregnant women with GD have an increased chance of developing T2DM after delivery. They also have an increased risk of developing hypertensive disorders during pregnancy, characterizing the pregnancy as high-risk, a fact that demands greater care and follow-up to prevent possible complications and death.^4^ ^5^ ^6^ ^7^ ^8^

Considering the increasing presence of the main risk factors for the development of GD in women of childbearing age and the fact that pregnancies associated with GD are characterized as high-risk, therefore requiring more caution and attention, the present research aimed at comparing the maternal and fetal outcomes of parturients with and without a diagnosis of GD.

## Methods

This was a case-control study including parturients with and without a GD diagnosis, who delivered between May and August 2018 at Hospital Nossa Senhora da Conceição (HNSC), located in the city of Tubarão, in the state of Santa Catarina, Southern Brazil.

The HNSC has an obstetric center that is a reference for high-risk management in the South of Santa Catarina, and ∼ 200 deliveries are performed there monthly. Using the Statcalc function of the Epi Info 3.5.4 (CDC, Atlanta, GA, US) software, and with the purpose of conducting a case-control study in the ratio of cases: 1:2 controls, we assumed a prevalence of ∼ 19% of obesity as a risk factor for the development of GD in the general population, considering that the incidence of gestational diabetes mellitus (GDM) in obese pregnant women is three times higher than in the general population. The sample size was calculated as 140 parturients (47 cases and 93 controls).^9^ ^10^

The present study included parturients with and without a GD diagnosis, who delivered at the HNSC between May and August of 2018; after being informed, they accepted to participate in the study and signed an Informed Consent Form (ICF). Parturients with type 1 DM (DM1) and T2DM were excluded. The diagnostic criteria of GD followed the Consensus on Gestational Diabetes: 2017 Update.^10^ The diagnosis of GD is established with glycemic values of fasting glucose between 92–125 mg/dL or between 24–28 weeks of gestational age (GA), in the oral glucose tolerance test (OGTT) 75 g with glycemic value ≥ 180 mg/dL after 1h of the overcharge, and between 153–199 mg/dL 2h after the overcharge.^11^

Data collection was performed from May to August 2018. The data source used was primary, in which the researchers contacted the parturients admitted to the HNSC, who, through the ICF, agreed to participate; and secondary, in which the researchers sought information related to the newborn (NB) in electronic medical records. The controls were selected among the puerperae without GD in the same period.

The researchers visited the hospital daily and contacted all of the parturients admitted to explain the research and to apply the questionnaire to those who accepted to participate.

The instrument used was a self-administered questionnaire and a protocol developed by the researchers containing the variables of interest, such as: age (years); weight (kg); weight gain (body mass index [BMI] values); height (cm); previous BMI (kg/m^2^); method of diagnosis; GA at diagnosis (in weeks); family history of DM; previous history of GD; presence of gestational hypertension; delivery route (vaginal, cesarean section); GA at delivery (weeks); fetal weight at birth (kg); Apgar score at 1 and 5 minutes; hypoglycemia at birth; RDS; fetal death; and the NB gender (female, male). The pregestational and current (time of delivery) weight and height values were mentioned by the participants, considering the impossibility of the researchers to be present full time to carry out the measurements. The BMI was calculated by the researchers (weight/height^2^), and the weight gain was calculated by subtracting the BMI from the last gestational weight of the pregestational BMI, and categorized as normal (BMI < 25 kg/m^2^), overweightness (BMI 25–29.99 kg/m^2^), and obesity (BMI ≥ 30 kg/m^2^).

In the present study, excess weight was characterized as overweightness and obesity, and gestational hypertension was characterized by decompensated chronic hypertension during pregnancy or hypertension developed during pregnancy. The GA at delivery was defined as premature when the delivery occurred before 37 weeks. Macrosomia was defined as birth weight ≥ 4 kg in term deliveries or > the 90th percentile for the GA. Hypoglycemia at birth was defined by the pediatrician. The researchers had no access to the absolute blood glucose levels in cases of NBs with neonatal hypoglycemia.

The collected data were inserted and stored in a database created with the help of the software Epi Info, version 3.5.4. The data analysis was performed using this software and complemented with the Statistical Package for the Social Sciences (SPSS, IBM Corp. Armonk, NY, US) software, version 20.0. Descriptive statistics were used as absolute numbers and percentages, measures of central tendency and dispersion. The bivariate analysis was performed using the Student *t* test for quantitative variables and the Chi-squared or Fisher exact tests for categorical variables, according to data suitability. Comparisons between cases and controls were expressed using odds ratio (OR), with a 95% confidence interval (95%CI) and a statistical significance level of 5%. The variables that presented *p*-values < 0.20 in the bivariate analysis were submitted to backward conditional multivariate logistic regression to evaluate the independent association with the outcome.

The present research followed Resolution 466/2012 of the Brazilian National Health Council, and was approved by the Ethics in Research Committee of Universidade do Sul de Santa Catarina (UNISUL) on March 28, 2018, under opinion number 2.569.614.

## Results

In the 140 women included in the present study (47 cases and 93 controls), the minimum age was 18 years old, and the maximum age was 44 years old. The mean age of the cases was 32.8 ± 6.4 years, which was statistically higher than that of the controls: 27.2 ± 6.9 years. The cases were more likely to be 35 years old or older (42.6%) compared with the controls (14%). Regarding the previous BMI, the cases presented more pregestational overweightness (72.3%) than the controls (50.5%). There was also a greater chance of obesity in the cases than in the controls (Table 1).

**Table 1:** Factors related to the development of gestational diabetes

Variables	Cases n = 47 (%)	Controls n = 93 (%)	OR	95%CI	*p*-value
Age ≥ 35 years	20 (42.6)	13 (14.0)	4.56	2.00–10.38	0.0002*
Overweightness	34 (72.3)	47 (50.5)	2.56	1.20–5.46	0.014*
Obesity	20 (42.6)	20 (21.5)	2.70	1.26–5.78	0.009*
FH T2DM	31 (66.0)	40 (43.0)	2.57	1.24–5.33	0.01*
PH GD	3 (6.4)	2 (2.2)	3.10	0.50–19.24	0.2098^∞^
	**Mean ± SD)**	**Mean ± SD)**			***p*** **-value**
Age (years)	32.80 ± 6.38	27.20 ± 6.29		−	0.0002^#^
Height (cm)	160.25 ± 7.84	161.50 ± 6.62		−	0.328^#^
Previous BMI (kg/m^2^)	29.90 ± 6.40	26.33 ± 6.12		−	0.0013^#^
Weight gain (BMI values)	4.46 ± 3.26	4.62 ± 3.26		−	0.79*

Abbreviations: 95%CI, 95% confidence interval; BMI, body mass index; FH T2DM, family history of type 2 diabetes mellitus; OR, odds ratio; PH GD, previous history of gestational diabetes, SD, standard deviation.

Notes: *Chi-squared test. ^∞^Fisher exact test. ^#^Student *t* test.

There was no significant difference in mean weight gain, previous history of GD, and height between cases and controls. The cases had a greater chance of having a positive family history of DM than the controls. The risk factors associated with the development of GD analyzed in the present study are described in Table 1.

In the conditional logistic regression, age emerged as an independent variable associated with the occurrence of GD in all models (Table 2). We verified by the forward method that both obesity and family history lost significance when placed together in a model that included age. This fact was due to the association between the 2 variables; family history of T2DM was present in 65% (26/40) of obese, women and in 45% (45/100) of nonobese women (*p* = 0.032). Furthermore, women older than 35 years of age were more obese (42% [14/33] than younger women (24.3% [26/107]; *p* = 0.044). Considering the previous BMI and age as continuous variables, both were associated with the occurrence of GD; but, when combined in the model, age prevailed as an associated factor. However, the adequacy measure of the model (R^2^ ≤ 0.21) indicated little explanation for the relationship between the surveyed variables and GD, suggesting that there were other factors influencing the outcome that were not evaluated in the present study.

**Table 2:** Association model of variables for gestational diabetes in a conditional logistic regression model

Variable	Initial adjustment model^a^		*p*-value	Final adjustment model^b^		*p*-value
	OR	95%CI		OR	95%CI	
Age ≥ 35 years	3.690	1.520–8.950	0.004	3.87	1.651–9.074	0.002
Overweightness	1.430	0.543–3.767	0.469	−	−	−
Obesity	1.710	0.645–4.555	0.280	2.08	0.920–4.707	0.079
FH T2DM	2.010	0.910–4.457	0.084	2.09	0.962–4.550	0.063
PH GD	2.880	0.384–21.614	0.304	−	−	−
Weight gain	0.981	0.868–1.108	0.754	−	−	−

Abbreviations: 95%CI, 95% confidence interval; FH T2DM, family history of type 2 diabetes mellitus; OR, odds ratio; PH GD, previous history of gestational diabetes.

Notes:^a^Initial model: step 1 of backward conditional logistic regression with all variables inserted. ^b^Final model: stage 4 of the regression process, after the removal of the variables weight gain, excess weight, and PH GD (in that order).

The mean GA at diagnosis was of 25.2 ± 8.51 weeks, with a minimum GA of 7 weeks, and a maximum GA of 38 weeks. The most prevalent method of diagnosis of GD was the OGTT, which established the diagnosis in 76.6% (36) of the cases, while 23.4% (11) were diagnosed by fasting glucose (FG).

The mean birth weight was significantly higher in the infants of mothers diagnosed with GD. Regarding the delivery route, no statistically significant difference was found between mothers with and without GD, and those who had GD also had no greater chance of having gestational hypertension than the controls.

There were no significant differences in the mean GA at delivery, and 22 out of the 140 (15.7%) parturients had preterm births. The mean Apgar score at 1 minute ranged from 1 to 10, while at 5 minutes it ranged from 7 to 10. One infant had RDS, and no deaths were observed in the overall cohort. The outcomes related to the development of GDs are described in Table 3.

**Table 3:** Outcomes related to the presence of gestational diabetes

Outcomes	Cases (*n* = 47) (%)	Controls (*n* = 93) (%)	*p*-value
Gestational hypertension	12 (25.50)	18 (19.40)	0.400*
Hypoglycemia at birth	05 (71.40)	02 (29.00)	0.01*
Macrosomia	17 (36.20)	10 (18.80)	0.00031*
Prematurity	06 (12.80)	15 (16.00)	0.495*
Cesarean section	28 (59.60)	47 (50.50)	0.311*
RDS	01 (2.12)	0 (0)	0.111^∞^
Death	0 (0)	0 (0)	−
	**Mean ± SD**	**Mean ± SD**	
Weight at birth (kg)	3.37 ± 0.64	3.08 ± 0.63	0.01^#^
GA at birth (weeks)	38.30 ± 2.15	38.33 ± 2.31	0.847^#^
Apgar score at 1 minute	8.20 ± 1.71	8.40 ± 1.27	0.424^#^
Apgar score at 5 minutes	9.40 ± 0.77	9.53 ± 0.75	0.665^#^

Abbreviations: GA, gestational age; RDS, respiratory distress syndrome; SD, standard deviation.

Notes: *Chi-squared test. ^∞^Fisher exact test. ^#^Student *t* test.

Out of the 47 women diagnosed with GD, 87.2% (41) underrwent some type of treatment, including diet alone (27/57.4%), insulin alone (04/8.5%), diet plus oral medication (08/17%), and diet plus insulin (02/4.3%). A total of 12.8% (06) of the cases underwent no treatment despite medical advice to do so. None of the participants used only oral medication. There were no significant differences regarding submission or not to treatment and the occurrence of maternal and fetal outcomes. A comparison between submission or not to treatment in relation to the outcomes is shown in Table 4.

**Table 4:** Comparison of pregnancy outcomes in relation to treatment or not of gestational diabetes

	Treatment n = 41 (%)	No treatment n = 6 (%)	*p*-value^∞^
Macrosomia	13 (31.70)	04 (66.70)	0.115
Gestational hypertension	10 (24.40)	02 (33.30)	0.486
Prematurity	05 (12.20)	01 (16.70)	0.581

Note: ^∞^Fisher exact test.

Observing the type of treatment to which the women with GD were submitted and the related factors, we found no significant differences regarding the use of insulin in relation to maternal age, previous BMI, and overweightness. Regarding pregnancy outcomes, the use of insulin was not associated with the occurrence of preterm delivery, and although cesarean sections were performed more frequently in women taking insulin, this finding was not significant. The evaluation of the other types of treatment in regard to the related factors was limited by the small sample of women with GD who underwent treatment. The relationship between the use of insulin for treatment in women with GD, the related factors, and pregnancy outcomes is shown in Table 5.

**Table 5:** Relationship between insulin use for the treatment of women with gestational diabetes, related factors, and pregnancy outcomes

	Insulin use		*p*-value
	Yes (*n* = 6) (%)	No (*n* = 41) (%)	
Age ≥ 35 years	16.7	46.3	0.169*
Overweightness	83.3	70.7	0.46
Preterm delivery	16.7	12.2	0.6
Cesarean section	83.3	56.1	0.2

Notes: *Chi-squared test. ^∞^Fisher exact test.

Out of the NBs with macrosomia (27), 17 (63%) were born to mothers with GD, and 10 (10.8%) were infants of women without GD (*p* = 0.0003). Regardless of the presence of GD, macrosomia was a more common event in males (19/72 [26.4%]) than in females (8/68[11.8%]); *p* = 0.028. In the same way, hypoglycemia was an outcome more frequent among infants of mothers with GD (5/47 [10.6%]) than among those of mothers without the disease (2/93 = 2.2%); *p* = 0.042.

## Discussion

The literature shows that age > 35 years is a risk factor for the development of GD. Alves et al^11^ found GD to be the second most frequent complication in the pregnancies of women older than 35 years, representing 17% (*n* = 430 women) of the complications found. In a case-control study^12^ (*n* = 206 cases and *n* = 286 controls) in China, older maternal age was also associated with risk of developing GD. Findings in Southern Brazil of two cohorts^13^ of women with GD, separated by 20 years, showed a mean age of 31 ± 7 years (*n* = 375) and 30 ± 6 years (*n* = 216). This relationship between age and GD was evident in the present study, in which the mean age of the cases was higher than that of the controls.^12^ ^13^ ^14^

The cases had a greater chance of being overweight and obese than the controls. Feleke^14^ also relates the previous BMI and a higher chance of developing GD in a case-control study developed in Ethiopia in the period from January to June 2016, in which the cases had 2.96 times greater chance of having a BMI ≥ 25 kg/m^2^ (cases: *n* = 568; controls: *n* = 1,702). Miao et al,^15^ in a retrospective analysis of nulliparous women diagnosed with GD (*n* = 832), showed that, in general, 21.4% (*n* = 178) of the women were obese or overweight, and 35.2% (*n* = 298) presented excessive weight gain during pregnancy. In the present study, there was no significant difference in the mean weight gain between cases and controls. This is probably due to the fact that, in the present study, weight values were referred by the parturients, and were not measured.^15^ ^16^

Corroborating the data obtained in the present research, a retrospective study in China^17^ comparing women with GD (*n* = 996) and without GD (*n* = 996) showed that a positive family history of DM was significantly more frequent in the cases than in the controls (15.6% versus 2.4% respectively). In relation to the presence of previous history of GD, differently from what was found in the present study, Bhat et al^16^ found that the cases (*n* = 300) were 5.3 times more likely to have a previous history of GD than the controls (*n* = 300). This difference is, perhaps, due to the fact that in their Study, the number of cases was at least one-sixth times lower.^17^ ^18^

In patients with GD, the mean height of the cases was above the one considered as a predisposing factor for GD (150 cm). However, in a cohort study^18^ performed in the city of Pelotas, Southern Brazil, in which 2.95% of the 4,243 participants reported GD, the mean height was 158.6 cm and a greater height emerged as a protective factor against the development of GD.^19^

The mean GA at diagnosis is within the period of highest physiological insulin resistance in pregnancy, and the one recommended for GD screening (24–28 weeks). The most prevalent method of diagnosis of GD was the OGTT, probably due to the fact that most diagnoses were established in the period of higher hyperglycemia risk, in which the investigation is performed through the OGTT and not the FG. In a study^19^ that evaluated hyperglycemia during pregnancy, 16.3% (*n* = 4,053) of the sample had a GD diagnosis, with a mean GA at diagnosis of 21 weeks, varying from 15 to 27 weeks. Furthermore, in a prospective study^20^ (cases: *n* = 35; controls: *n* = 465), 11.4% (*n* = 4) of the sample had their diagnosis in the initial 16 weeks, and 88.6% (*n* = 31), in 24–28 weeks.^20^ ^21^

Miranda et al,^22^ in a case-control study (cases: *n* = 201; controls: *n* = 201), found hypoglycemia at birth to be the most frequent complication of GD, and identified hypoglycemia and RDS as important causes of neonatal morbidity; in relation to the birth weight of the NBs, there were no statistical differences. In the present study, the mean birth weight and rate of neonatal hypoglycemia were significantly higher in infants of mothers with GD, and, when considering RDS, the same did not occur in the study by Miranda et al.^21^ The lower rate of RDS in our study was possibly associated with the fact that most deliveries occurred at term, with lesser chance of problems related to delayed pulmonary maturity.^22^

The proportion of cesarean sections in the present study was similar to that found in a retrospective cohort study^22^ with women with GD diagnosis (*n* = 703) in a hospital in the city of Joinville, Southern Brazil: 52.2% (*n* = 367) had cesarean sections as the delivery route.^23^

Zanrosso et al,^23^ in a descriptive study (GD cases: *n* = 86) conducted in the city of Canoas, Southern Brazil, suggested a greater frequency of prematurity in women with GD; 3 deaths were recorded in the study. Likewise, in a retrospective cohort^24^ (cases: *n* = 255; controls: *n* = 267), women with GD showed twice the risk of delivering preterm infants. In addition, the study showed that the association between GD and a low Apgar score at 1 and 5 minutes was not significant. In the present study, there were no significant differences regarding the Apgar score and the mean GA at birth in infants of mothers with and without GD. The absence of neonatal deaths in our study is probably related to the current greater care dedicated to pregnant women with GD and also to therapy individualization, which reduces unwanted GD outcomes.^24^ ^25^

As in the present study, a transversal study^26^ (*n* = 159 pregnant women) conducted in the city of Maceió, Northeastern Brazil, in which the outcomes of interest were GD and gestational hypertensive syndrome (GHS), observed no association between GD and gestational hypertension, since none of the diseases occurred concomitantly.^26^

According to the Hyperglycemia and Adverse Pregnancy Outcomes (HAPO) study,^26^ there is a relationship between maternal hyperglycemia and the occurrence of unwanted pregnancy outcomes, which are minimized with treatment. Therefore, treatment should be undergone by all pregnant women with an established diagnosis of GD to reduce adverse maternal-fetal outcomes. Nevertheless, adherence to treatment was not found in 100% of the cases in the present study. As expected, the only treatment that most pregnant women with GD underwent was a diet. A retrospective study^28^ (cases: *n* = 799; controls: *n* = 2,843) in 3 maternity hospitals in Chennai, India, showed similar results: most women underwent a diet as the only treatment 42.2% (*n* = 338) or diet associated with insulin 57.1% (*n* = 457), while only 0.5% (*n* = 4) used oral medication.^27^ ^28^ ^29^

Regarding the relationship between outcomes and treatment in GD patients, lack of treatment is known to increase the risk of unwanted outcomes. In the present study, undergoing treatment did not interfere in the occurrence of macrosomia, hypertension, or prematurity. This differs from the observation of a systematic review,^29^ which found a lower prevalence of preeclampsia and a lower birth weight among pregnant women who underwent treatment. Regarding GA at delivery, there were no significant differences. In a retrospective cohort study^30^ (*n* = 705 women with GD) performed in the city of Joinville, Southern Brazil, in which all women underwent some kind of treatment, 10.22% (*n* = 72) of the treated pregnant women developed hypertensive pregnancy disease, and 4.8% (*n* = 34) of the infants were premature.^28^ ^30^ ^31^

Also in the aforementioned study,^30^ observing the type of treatment performed and the factors related to the occurrence of GD or the outcomes, insulin use was related to a lower probability of preterm delivery 7.1% (*n* = 50), and the type of treatment performed did not interfere with the delivery route, reflecting the same finding observed in the present study, except that the study in question found a higher rate of cesarean sections among parturients taking insulin. Watanabe et al^31^ also found a higher rate of cesarean sections among women treated with insulin; in addition, 40% (*n* = 4) of them were overweight, and 10% (*n* = 1) had a BMI 30 kg/m^2^ before pregnancy, which was not significant.^31^ ^32^

Regarding the maternal age and insulin use, no significant differences were found either. Differently, a retrospective study^32^ (*n* = 612 GD cases) showed that the mean age of the women who used insulin was 31.4 years versus 30.9 years for those who did not use it. Furthermore, women who took insulin had a higher BMI than those who did not require it (28.3 ± 7.00 kg/m^2^). In the present study, overweight parturients had more chance of using insulin than those who were not overweight, a finding with no statistical significance.^33^

According to Ribeiro et al^33^ in a case-control study (cases: *n* = 149; controls: *n* = 711), the prevalence of NBs with macrosomia ranged from 5 to 20%. In addition, the study showed that male NBs had 3.33 times more chance of being macrosomic when compared with female NBs. This finding was also observed in the present study. The predominance of macrosomia in males may be associated with the fact that, during the third trimester, male fetuses tend to gain more weight than female ones.^34^ ^35^

A limitation of the present study was the reduced number of cases, which hinders the generalization of the findings to other populations. Furthermore, specific information, like treatment adherence and body weight, could not be measured, and the self–reported data are subject to errors.

## Conclusion

The present study found maternal age, family history of DM2, obesity and pregestational overweightness as important factors related to a higher chance of developing GD. Regarding the neonatal outcomes, we found that the children of women diagnosed with GD had higher prevalence of fetal macrosomia and hypoglycemia at birth than the children of women who did not have GD. There was no significant difference between cases and controls regarding gestational hypertension, prematurity, cesarean section, neonatal RDS and neonatal death.
